# Antioxidant, neuroprotective and anti-inflammatory activity of *Curcuma longa* extracts: from green extraction to nanoemulsion

**DOI:** 10.3389/fnut.2025.1619725

**Published:** 2025-09-24

**Authors:** Adriana Perez, Hugo A. Martinez-Correa, Fabiãn Parada-Alfonso, Zully Jimena Suárez Montenegro, Diego Ballesteros-Vivas, Gerardo Álvarez-Rivera, Alejandro Cifuentes, Elena Ibáñez

**Affiliations:** ^1^Universidad Nacional de Colombia-Sede Palmira-Facultad de Ingeniería y Administración, Chapinero, Colombia; ^2^High Pressure Laboratory, Food Chemistry Research Group, Facultad de Ciencias, Departamento de Química, Universidad Nacional de Colombia, Bogotá, Colombia; ^3^Facultad de Ingeniería Agroindustrial, Universidad de Nariño, Ciudad Universitaria Torobajo, San Juan de Pasto, Colombia; ^4^Facultad de Ciencias, Departamento de Nutrición y Bioquímica, Pontificia Universidad Javeriana, Bogotá, Colombia; ^5^CRETUS, Department of Analytical Chemistry, Nutrition and Food Science, Universidade de Santiago de Compostela, Santiago de Compostela, Spain; ^6^Foodomics Laboratory, Institute of Food Science Research (CIAL) (CSIC-UAM), Madrid, Spain

**Keywords:** curcuminoids, antioxidant activity, acetylcholinesterase, lipoxygenase, anti-inflammatory, supercritical fluid extraction, green extraction

## Abstract

*Turmeric rhizomes* are rich in bioactive compounds like curcuminoids, offering antioxidant and anti-inflammatory properties. It is a medicinal plant aiding in the prevention of neurological, cardiovascular, and metabolic diseases. In the present study, the sequential extraction of turmeric (*Curcuma longa L*.) combining supercritical fluid extraction (SFE) followed by ultrasound-assisted extraction (UAE) has been described for the first time to improve the yield and bioactivity of the different fractions. In the SFE stage, the maximum oil extraction yield (4.0%) was obtained; under these conditions, maximum bioactives’ content was obtained. In the UAE stage, the total phenolic content (TPC), curcuminoids, antioxidant capacity (DPPH, ABTS), inhibitory activity against the acetylcholinesterase enzyme (AChE), and against the lipoxygenase enzyme (LOX) were analyzed, together with a complete profile by UHPLC-qTOF-HRMS in order to tentatively identify the compounds responsible of the observed bioactivities. The UAE process resulted in a maximum TPC of 181.51 mg GAE/g DE, CUR of 604.40 mg/g DE, highlighting a high inhibitory potential for AChE (IC_50_: 5.21 μg/mL) and LOX (IC50: 17.96 μg/mL). A nanoemulsion of the obtained extract showed no significant changes after 15 days, indicating good stability and homogeneous particle distribution. The SFE + UAE combination enhances yield and concentration of bioactive compounds like phenols and curcuminoids, optimizing turmeric extract’s neuroprotective and anti-inflammatory potential and providing fractions with specific composition of bioactives, thus targeting different health benefits. In this sense, both fractions could be advantageously employed to develop nutraceuticals and/or functional foods with specific health benefits.

## Graphical abstract

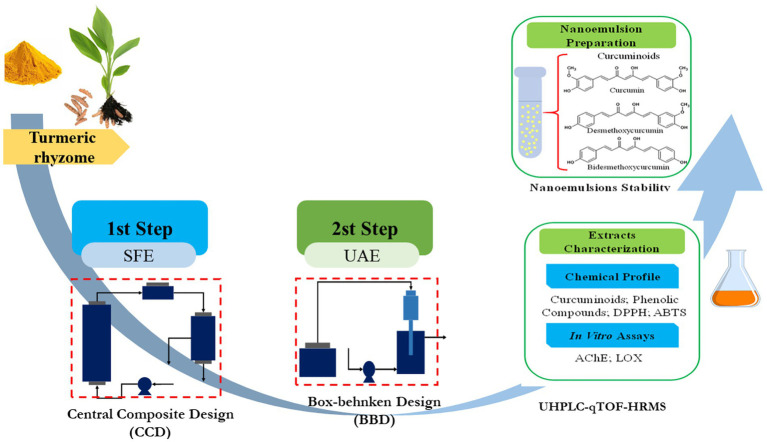

## Highlights

- The combined supercritical CO₂ and ultrasound method yields high curcuminoid content.
- AChE assays demonstrate turmeric extracts’ strong neuroprotective potential.
- LOX assays revealed potent anti-inflammatory activity in turmeric extracts.

## 1 Introduction

Bioactive compounds present in various food matrices and medicinal plants are gaining increasing relevance in human health, as recent research has shown that they can aid in the treatment and delay the onset of chronic diseases (1, 2). Alzheimer’s disease affects approximately 45 million people worldwide and is expected to reach 80 million by 2050 (3). It is characterized by cognitive dysfunction, neuroinflammation, and oxidative stress (4). Acetylcholine (ACh) is the primary neurotransmitter in the brain, and its reduction has been linked to cognitive dysfunction. For this reason, acetylcholinesterase (AChE) enzyme inhibitors are commonly used in the treatment of diseases such as dementia and Alzheimer’s disease (3, 5, 6). The lipoxygenase (LOX) enzyme has been associated with neuroinflammation and may be a key mediator in neurodegenerative diseases. It is estimated that LOX enzyme inhibition could generate new alternatives for the treatment of Alzheimer’s disease and other neurodegenerative disorders (3, 5).

Recent studies indicated that *Curcuma longa* rhizomes could be a natural resource with high potential in bioactive compounds, exhibiting antioxidant, anti-inflammatory, neuroprotective, antimutagenic, antiallergic, and anticancer properties, among others (7–9). Numerous studies suggested that turmeric is a medicinal plant that could aid in the prevention of neurological, cardiovascular, and metabolic diseases, such as diabetes (10–12). Turmeric rhizomes contain essential oils, phenolic compounds and curcuminoids. These bioactive compounds vary in quantity depending on genotype, variety, geographical conditions, cultivation conditions, and seasonality (13, 14). However, curcuminoids have limitations for food and pharmaceutical applications, such as low water solubility, low bioavailability, rapid degradation under neutral or alkaline pH, and photodegradation when exposed to light (7, 15). Given this scenario, nanoemulsions, which are colloidal systems, play an important role as they can encapsulate, protect, release, enhance solubility and bioavailability of bioactive compounds (16–18).

Conventional extraction methods (e.g., solvents, maceration, Soxhlet) have significant limitations. They employ large volumes of toxic organic solvents, which causes environmental pollution and makes disposal difficult. In addition, the high temperatures used in solvent extraction and separation increase the risk of degradation of bioactive compounds, compromising the quality of the extract. The extraction of bioactive compounds using solvents such as supercritical carbon dioxide (Sc-CO_2_) and ethanol, through techniques like SFE and UAE, has emerged as a viable and environmentally friendly alternative to conventional extraction processes. These green technologies have proven to be effective in recovering important bioactive compounds from plants, seeds, fruits, and vegetables (5, 19). The SFE + UAE combination has not been previously studied for extraction of bioactive compounds from curcuma. This novel integrated approach, coupled with analytical and *in vitro* methods, is proposed as a powerful strategy for identifying new components and evaluating their biological activities, which could be associated with potential human health benefits (5, 20). Therefore, this study aims to (1) optimize sequential SFE + UAE for turmeric extracts, (2) evaluate their bioactivities, and (3) assess nanoemulsion stability.

## 2 Materials and methods

### 2.1 Plant raw material

*Curcuma longa* rhizomes were obtained from agroecological crops located in Valle del Cauca, Colombia (latitude = 3° 43′28” N, longitude = 76° 16′02” W). The rhizomes were washed and dried (40 °C, 15 h). The dried and ground rhizomes had an average particle diameter of 0.402 mm and an approximate moisture content of 14.38% (wb). The samples were stored under refrigeration (5 °C) in the absence of light and oxygen (21).

### 2.2 Reagents

Ethanol 99.5% w/w (Millipore Direct-Q 3 UV, Millipore Corporation, USA), analytical-grade hexane (J. T. Baker), and analytical standard curcumin (75% purity) were purchased from Sigma-Aldrich (USA). Acetylcholinesterase (AChE) from *Electrophorus electricus* (electric eel) type VI-S and 2,2′-azino-bis (3-ethylbenzothiazoline-6-sulfonic acid) (ABTS•+) were obtained from Sigma-Aldrich (USA). Trizma hydrochloride (Tris–HCl), bovine serum albumin (BSA), monopotassium phosphate (KH_2_PO_4_) ≥ 99.0%, disodium phosphate (Na_2_HPO_4_) ≥ 99.0%, potassium persulfate (K_2_S_2_O_8_) ≥ 99.0%, sodium carbonate (Na_2_CO_3_) 99.0%, fluorescein sodium salt, quercetin, cholesterol, and linoleic acid were obtained from Sigma-Aldrich (Spain). 7-Fluorobenzofurazan-4-sulfonamide (ABD-F) 98% was purchased from Alfa Aesar (Japan); (±)-6-Hydroxy-2,5,7,8-tetramethylchroman-2-carboxylic acid (Trolox) > 97% was purchased from Sigma-Aldrich (Denmark). Acetylthiocholine iodide (ATCI) ≥ 98.0% and lipoxygenase from *Glycine max* (soybean), Type 1-B, were obtained from Sigma-Aldrich (United Kingdom). HPLC-grade ethanol (EtOH) was purchased from VWR Chemicals (Spain), and galantamine hydrobromide >98.0% was purchased from TCI Chemicals (Japan). Ultrapure water (18.2 MΩ cm) was obtained from a Millipore system (USA).

### 2.3 Extraction of curcuma compounds and statistical analysis

**Figure 1:**
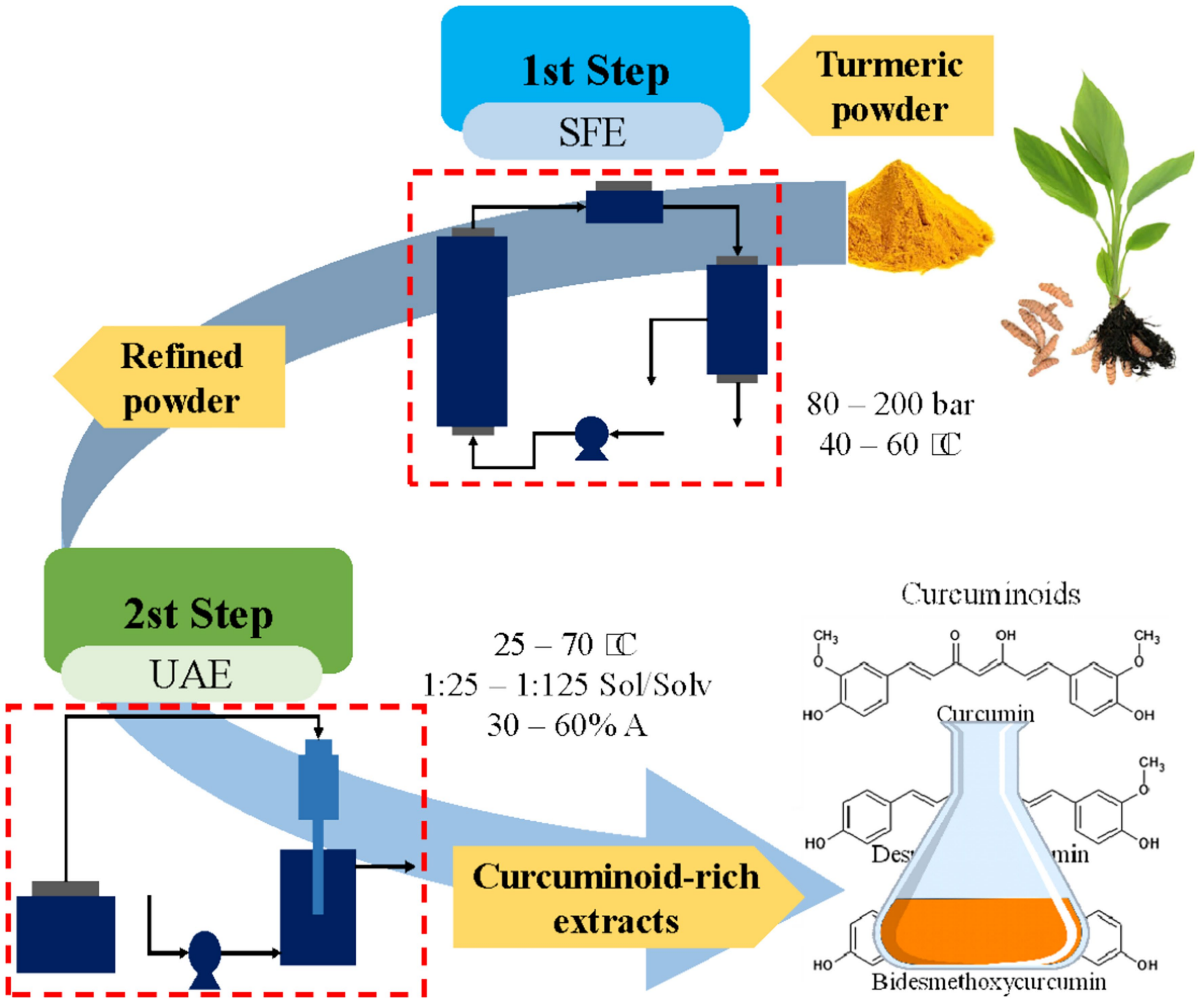
Sequential extraction of biocompounds from curcuma obtained through green technologies.

The SFE + UAE sequential extraction was studied using the residue from the supercritical fluid extraction step (that provided the highest volatile oil extraction yield) as a biomass for the UAE step (Figure 1).

#### 2.3.1 Stage 1

The SFE stage was performed to extract a significant portion of the low-polarity fraction of turmeric (essential oils) with supercritical CO_2_, aiming to reduce its characteristic odor caused by some volatile compounds. A 3-level-2-factor central composite design (CCD) approach-based response surface methodology (RSM) analysis was proposed (22). The CCD was selected to estimate first and second-order terms of the model since it gives information about the possible curvature (by the use of the axial points) of the response variables. In this study, the selected factors were pressure (80, 140, and 200 bar) and temperature (40, 50, and 60 °C), while the response variable was oil yield (%) (Supplementary Table S1). These pressure and temperature values were obtained from previous studies (23, 24). A total of 13 experimental runs were performed, comprising five center point replicates for the assessment of experimental error, as well as four axial and four factorial design points.

#### 2.3.2 Stage 2

In the UAE stage, 99.5% ethanol was used due to its designation as a green, safe (GRAS, generally recognized as safe), and non-toxic solvent with low cost and high recovery. A Box–Behnken design (BBD, 3-factor, 3-level) was employed to optimize UAE. The BBD was selected in this particular case since 3 factors were tested and it requires fewer runs than CCD for the same number of factors; moreover, all the design points are within the operating limits, with no axial points. The BBD included 17 randomized experiments, with five replicates at the central point. The coded and actual levels of the experimental variables, along with the response variables, are presented in Supplementary Table S3. The study variables were temperature (T, 40, 55, and 70 °C), solid/solvent ratio (Sol/Solv, 1:25, 1:35, and 1:45), and amplitude (A, 40, 60, and 80%). These values were obtained from previous studies (25). The response variables included total phenolic content (TPCmg GAE/g DE), curcuminoid content (mg/g DE), antioxidant activity (DPPH and ABTS: μmol ET/g DE), acetylcholinesterase inhibitory activity (AChE, μg/mL), and anti-inflammatory activity (lipoxygenase enzyme inhibition, LOX) (μg/mL). Ethanol is an effective, green, and safe solvent for curcuminoids extraction, its low-temperature operation prevents thermal degradation, offering economic and environmental benefits, including low cost and high recovery.

For stages 1and 2, analysis of variance (ANOVA) was applied using the significance level (*p* ≤ 0.05), F-statistic, and the coefficient of determination (R^2^) and the lack-of-fit to assess the relevance of each factor in the fitted models and to evaluate the adequacy of the model fit in each case. Among the different statistical tools, the lack-of-fit can help validating the adequacy of the model, identifying potential issues with the experimental design or data collection process and improving the model by incorporating additional terms. Design Expert® software version 13 (Stat-Ease, Inc., Minneapolis, MN, USA) was used for the experimental design, optimization and statistical analysis.

### 2.4 Extraction methods used as reference

#### 2.4.1 Essential oil extraction by hydrodistillation

Turmeric sample (30 g) was placed in a 500 mL round-bottom flask. Then, 200 mL of distilled water was added to the flask and heated to boiling. This extraction process was carried out for a period of 3 h (26). The extracted oil was stored under refrigeration (5 °C).

#### 2.4.2 Soxhlet extraction

Extracts were obtained with ethanol (99.5% w/w). For this, 12 g of the sample was used in the Soxhlet apparatus, with the solvent for 4 h (21, 22). After complete extraction, the solvent was removed by rotary evaporation. The solvent-free extracts were stored under refrigeration (5 °C).

### 2.5 Turmeric extract nanoemulsion

The extract selected for nanoemulsion formulation was obtained from the (SFE + UAE) treatment, which provided an extract with a high concentration of curcuminoids, total phenolics, antioxidant activity (DPPH and ABTS), and *in vitro* biological activity (AChE and LOX inhibition).

#### 2.5.1 Nanoemulsion preparation

The nanoemulsions were prepared according to the conditions established in previous trials using the same raw material (27). The process consisted of two phases. In the oil phase, the oil was heated to a constant temperature of 80 °C. At this temperature, the extract and SPAN 80 were added, and the mixture was stirred for one hour. The aqueous phase was prepared in parallel to the oil phase. The aqueous phase contained NaN₃ as an antimicrobial agent (0.001% w/v) (24) at 80 °C, followed by the addition of Tween 80 until complete dissolution was observed. The ratio between Tween 80/SPAN 80 surfactants was 2:1, calculated taking into account their hydrophobic balances (HLB). This specific ratio was established based on previous tests. The combination of a lipophilic (SPAN 80) and a hydrophilic (Tween 80) is a common strategy in emulsion formulation to reduce the interfacial tension between the oil and aqueous phases and achieve a stable emulsion.

A once both phases were prepared, they were mixed using a high-speed homogenizer (Ultraturrax IKA, T18) at 140,000 rpm for 15 min. Subsequently, the emulsions were treated with ultrasound at 40% amplitude for 15 min, while maintaining a water circulation bath at 12 °C. Finally, the emulsions were stored under refrigeration (5 ± 1 °C) until characterization. The same procedure was repeated to prepare the control nanoemulsion, which used an analytical standard of curcumin (>75% purity, Sigma-Aldrich, USA).

#### 2.5.2 Characterization and stability of nanoemulsion

Particle size distribution (DLS), polydispersity index (PDI), Z potential and pH were measured for the nanoemulsion obtained. Z potential, DLS and PDI were measured using the technique of light dynamic (DLS) (Zetasizer Nano ZS, Malvern Instruments Ltd) at room temperature (25 °C) (28, 75). pH was recorded using a pH meter (Mettler Toledo, model FE20) calibrated with buffer solutions at pH = 4 and pH = 7, at 25 °C ± 1 °C.

### 2.6 Characterization of the extracts

#### 2.6.1 Extraction yield (%)

The yield was determined based on the mass of the dry extract (MEs) and the amount of turmeric used (MP) (Equation 1).


(1) $$\text{Extaction yield}\;(\%)=(\frac{\mathrm{MEs}}{\mathrm{MP}}).100$$

#### 2.6.2 Essential oil analysis

A gas chromatography–mass spectrometry (GC–MS) analysis was performed to identify the compounds present in turmeric oils. The analysis was conducted using a gas chromatograph (Agilent 5,977 single quadrupole GC/MSD, USA) equipped with an HP5ms column (30 m × 0.25 mm × 0.25 μm df). The oven temperature was initially maintained at 50 °C for 2 min, then programmed to 250 °C at a rate of 6 °C/min for 15 min. The mass spectrometer operated in electron impact mode (70 eV), with the ion source temperature set at 230 °C. Helium was used as the carrier gas, and the m/z ratio range was 30 to 500 (29). Compound identification was carried out using the NIST database library, and quantification was performed based on the percentage of area.

#### 2.6.3 Chemical profiling of UAE-stage extracts via UHPLC-qTOF-HRMS analysis

Chromatographic separation was performed on an Agilent 1,290 UHPLC system (Agilent Technologies, Santa Clara, CA) equipped with a reversed-phase column (Zorbax Eclipse Plus C18, 2.1 × 100 mm, 1.8 μm; Agilent Technologies) maintained at 30 °C. A 5.0 μL aliquot of sample was injected, and separation was achieved using a binary solvent system consisting of (A) 0.01% (v/v) formic acid in water and (B) acetonitrile (ACN). The gradient elution program was set as follows: 0–7 min (0–30% B), 7–9 min (30–80% B), 9–11 min (80–100% B), 11–13 min (100% B), followed by re-equilibration to initial conditions (14 min, 0% B), with a constant flow rate of 0.5 mL/min.

The UHPLC system was coupled to an Agilent 6,540 qTOF mass spectrometer via an orthogonal electrospray ionization (ESI) source operating in negative-ion mode. Key MS parameters were optimized as follows: capillary voltage, 4,000 V; nebulizer pressure, 40 psi; drying gas flow rate, 10 L/min; gas temperature, 350 °C; skimmer voltage, 45 V; and fragmentor voltage, 110 V. Full-scan MS (50–1,100 m/z) and data-dependent MS/MS (50–800 m/z) modes were employed for structural elucidation.

Post-acquisition data processing was conducted using Agilent MassHunter Qualitative Analysis (v. B.08.00). Tentative compound identification was based on accurate mass measurements, isotopic distribution analysis, MS/MS fragmentation patterns, and database searches. Additional filtering strategies—including mass defect, diagnostic fragment ions, neutral loss, and background subtraction—were applied to enhance annotation reliability.

#### 2.6.4 Total phenolic content

The method described by Singleton et al. (30) was used, adapted to a 96-well microplate format. The calibration curve required for this methodology was prepared using a gallic acid standard (purity >99%). A volume of 60 μL of the sample at the appropriate concentration and 60 μL of Folin–Ciocalteu reagent (FCR) were added to all wells, followed by 180 μL of sodium carbonate solution. The microplate was then incubated at 30 °C for 30 min. Subsequently, the plate was vortexed, and the samples were read at 750 nm using a microplate reader (Biotek Elx 800, USA). The total phenolic content (TPC) results were expressed as milligrams of gallic acid equivalents per gram of dry extract (mg GAE/g DE).

#### 2.6.5 Curcuminoid content

UAE extracts were subjected to rotary evaporation (40 °C for 15 min.) to obtain dry extracts. For the measurement, 5 mg of dry extract was dissolved in a 5 mL flask and completed with methanol (99.9% purity, Merck). The samples were then analyzed using a spectrophotometer (Genesys 10S UV–VIS, Thermo Fisher Scientific Inc., USA) at wavelength of 425 nm. The calibration curve was prepared using an analytical standard of curcumin (75% purity, Sigma-Aldrich, USA). The curcuminoid content was expressed as mg/g DE. Curcuminoid Content for UAE extracts.

### 2.7 Antioxidant activity

#### 2.7.1 DPPH method

The free radical scavenging capacity against 2,2-diphenyl-1-picrylhydrazyl (DPPH) was evaluated using a 96-well microplate. A volume of 60 μL of the samples and 140 μL of DPPH solution were added to the wells, followed by vortex agitation. The plate was incubated at room temperature for 30 min. Spectrophotometric readings were taken at 515 nm using a microplate reader (Biotek Elx 800, USA). The results were expressed as micromoles of Trolox equivalents per gram of dry extract (μmol TE/g DE).

#### 2.7.2 ABTS method

The free radical scavenging capacity against ABTS [2,2′-azinobis-(3-ethylbenzothiazoline-6-sulfonic acid)] was evaluated. A volume of 60 μL of the samples was added to the microplate wells, followed by 240 μL of the ABTS radical cation solution. The microplate was vortexed before measuring the samples at 750 nm using a microplate reader (Biotek Elx 800, USA). The results were expressed as micromoles of Trolox equivalents per gram of dry extract or dry sample (μmol TE/g DE).

### 2.8 *In vitro* biological activities

#### 2.8.1 *In vitro* acetylcholinesterase (AChE) inhibition activity

The inhibitory capacity of the extracts against AChE enzyme was evaluated based on the method of Ellman (31), modified by fluorescent enzymatic kinetics using ABD-F as a fluorescent probe, as described by Sánchez-Martínez et al. (32, 76). The Michaelis–Menten constant (KM value) was previously measured to determine the substrate concentration at which the reaction rate is half of the maximum velocity. A stock concentration of AChE was prepared in Tris–HCl buffer (pH 8.0, 150 mM) with the addition of 0.1% BSA to maintain the stability of the stock solution before use. Galantamine and a well without extract sample were used as positive and negative controls, respectively, for the assay reaction. Kinetic fluorescence measurements were performed in a microplate reader at an excitation wavelength (λex) of 389 nm and an emission wavelength (λem) of 513 nm, with a runtime of 15 min at 1-min intervals and a temperature set to 37 °C to determine the average velocity (Vmean) of the enzymatic reaction.

#### 2.8.2 *In vitro* anti-inflammatory activity assay

The inhibitory activity against the enzyme lipoxygenase (LOX) was measured using a fluorescence-based enzymatic kinetic method with fluorescein as a probe. The Michaelis–Menten constant (KM value) was previously determined to establish the substrate concentration at which the reaction rate reaches half of the maximum velocity (29). Quercetin (0.08 mg/mL) and a well without an extract sample were used as positive and negative controls, respectively, for the assay reaction. Kinetic fluorescence measurements were performed in a microplate reader at an excitation wavelength (λex) of 485 nm and an emission wavelength (λem) of 530 nm, with a runtime of 15 min at 1-min intervals and a temperature set to 25 °C to determine the average velocity (Vmean) of the enzymatic reaction.

#### 2.8.3 Scanning electron microscopy (SEM)

The analysis was performed using a Quorum Q150R ES (USA) device with a sputter voltage of 60 mA and a sputter time of 40 s. Samples were dried and coated with 99.99% gold, and their surfaces were observed at 500 × magnification. SEM images were acquired using a Fei Quanta 200 (USA) microscope, operating at an acceleration voltage of 15 kV under high vacuum. SEM analyses were conducted on untreated turmeric (control), turmeric subjected to SFE, turmeric subjected to UAE, and turmeric subjected to sequential extraction (SFE + UAE).

#### 2.8.4 Fourier transform infrared spectroscopy (FTIR)

Measurements were performed using a Shimadzu IRTracer-100 Fourier transform infrared (FTIR) spectrometer equipped with a DLATGS detector and a He/Ne laser, operating within a wavenumber range of 400 to 4,000 cm^−1^. Powdered samples were analyzed using the transmission method by preparing KBr pellets (2.5 mg of sample in 150 mg of KBr), without any modifications to the sample. FTIR measurements were conducted on untreated turmeric (control), turmeric subjected to SFE, turmeric subjected to UAE, and turmeric subjected to sequential extraction (SFE + UAE).

### 2.9 Statistics

To assess how the extraction factors influenced the yield, concentration of bioactive compounds, and bioactivity of the extracts, we conducted three independent trials (*n* = 3). We used ANOVA (*α* = 0.05) and Tukey’s test in Design Expert® software version 13 (Stat-Ease, Inc., Minneapolis, MN, USA) to identify any significant differences between the treatments. All data are presented as the mean ± standard deviation.

## 3 Results and discussion

### 3.1 Curcuma oil extraction by SFE

Results of the experimental design are shown in Supplementary Table S1. The analysis of variance (Supplementary Table S2) indicates that both pressure and temperature had a significant effect, as well as their interaction (*p* < 0.05). The obtained quadratic model did not show a lack of fit (*p* > 0.05), making it suitable for predicting future supercritical fluid extraction (SFE) yield values. The values of R^2^ = 0.9304, R^2^adj = 0.9014 and R^2^pred = 0.6731, provided additional information regarding the model’s adequacy. To determine the optimal extraction yield response, Derringer’s desirability method was applied using Design Expert software. The predicted responses are converted into a dimensionless desirability value (D), ranging from 0 (undesirable) to 1 (fully desirable) (22), obtaining a high desirability value (*D* = 0.98) at conditions of 160 bar and 43 °C. These conditions were experimentally validated, achieving an average yield of 4.0%.

**Figure 2:**
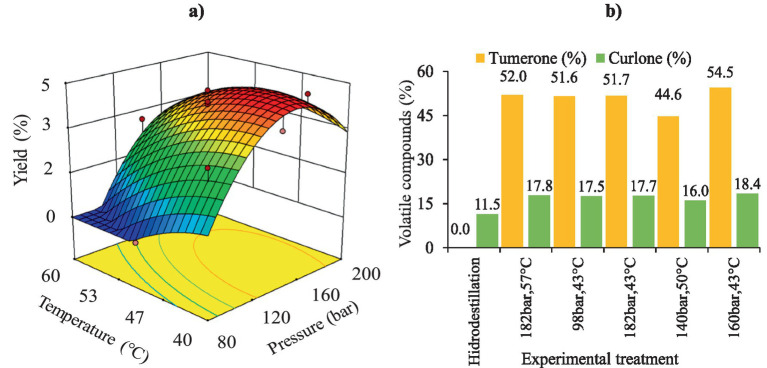
Extraction of turmeric oil using supercritical CO₂: **(a)** response surface of yield; **(b)** volatile compounds: Percentage of tumerone and curlone.

Figure 2a shows a negative effect, mainly at high temperatures (> 46 °C) and low pressures, on the extraction yield. This is due to a decrease in the density of Sc-CO₂ and, consequently, lower oil solubility (33). Similarly, Carvalho et al. (34) reported a decrease in oil solubility within the studied pressure range (80–200 bar), leading to low extraction yields. These results are very similar to those found in this study, where a correlation can be observed (Figure 2a and Supplementary Tables S1, S2) between the increase in pressure (up to 166 bar), the increase in solvent density and the enhancement of oil solubility in Sc-CO₂. The positive effect of SFE may be related to the characteristics of the raw material and some overlapping phenomena of vapor pressure over solvent density (33, 35, 36).

However, the results also indicate that when both pressure and temperature increase, extraction yields are not very high. The effect of temperature increase in SFE primarily depends on the working pressure. Increasing the temperature, at constant pressure, promotes two opposite effects: it reduces the solvent power of CO_2_ by a decrease in the density, and, on the other hand, it increases the vapor pressure of solutes, which can be more easily transferred to the supercritical phase. The balanced effect on the solubility of the solute in the supercritical solvent will, in fact, depend on the operating pressure. Near the critical pressure, the effect of fluid density is predominant, thus, a moderate increase in temperature leads to a large decrease in the fluid density, and therefore, to a decrease in solute solubility. However, at high pressures, the increase in the vapor pressure prevails, thus the solubility increases with the temperature. This is called a retrograde behavior of the solute solubility (35, 37).

#### 3.1.1 Volatile compounds obtained with supercritical CO₂

The volatile compounds present in the oils from five different treatments were analyzed: treatments with the highest extraction yield (T4 and T6, Supplementary Table S1), treatments with the lowest yield (T1 and T2, Supplementary Table S1), treatment under optimal conditions (TO: 160 bar and 43 °C), and treatment performed using hydrodistillation (HD) as a reference for conventional extraction A total of 50 compounds were identified as predominant in the five samples; however, semi-quantification was performed only for tumerone and curlone, as they were found in higher proportions (Table 1).

**Table 1:** Main constituents of turmeric oil measured by GC considering different SFE treatments (T1, T2, T4, T6 see Supplementary Table S1 for experimental conditions), optimum treatment (TO) and conventional hydrodistillation (Hd).

N°	Compound	T1 (%)	T2 (%)	T4 (%)	T6 (%)	TO (%)	Hd (%)
1	Caryophyllene	0.4	0.4	0.4	0.3	0.4	0.3
2	Benzene, 1-(1,5-dimethyl-4-hexenyl)-4-methyl-	1.7	2.3	1.7	2.1	1.3	2.6
3	1,3-Cyclohexadiene, 5-(1,5-dimethyl-4-hexenyl)-2-methyl-, [S-(R*, S*)]-	3.2	4.1	3.2	1.8	3.6	0.4
4	beta.-Bisabolene	0.6	0.8	0.6	0.6	0.6	0.7
5	Cyclohexene, 3-(1,5-dimethyl-4-hexenyl)-6-methylene-, [S-(R*, S*)]-	2.8	3.6	2.8	2.4	2.8	1.7
6	2-Methyl-6-(p-tolyl)hept-2-en-4-ol	0.9		0.9	1.0	0.8	1.9
7	Trans-Sesquisabinene hydrate	0.5	0.5	0.5	0.6	0.5	0.9
8	2-Methyl-5-((R)-6-methylhept-5-en-2-yl)bicyclo[3.1.0]hex-2-ene	1.8					
9	(S)-(−)-1-(p-Tolyl)ethylamine	2.1					
10	(E)-.gamma.-Atlantone	0.5	0.4	0.7	0.5	1.4	1.2
11	Tumerone	52	51.6	51.7	44.6	54.5	
12	Curlone	17.8	17.5	17.7	16.0	18.4	11.5
13	(Z)-.gamma.-Atlantone	1.6		1.4	1.0		
14	1-Butoxypropan-2-yl 2-methylbut-2-enoate	1.8					
15	Binapacryl	2.9					
16	Pinocamphyl angelate, iso-	2.2		2.6	2.8		
17	6-(2-Hydroxy-4-methylphenyl)-2-methylhept-2-en-4-one	1.4		1.3		0.8	
18	(1R,4R)-1-methyl-4-(6-Methylhept-5-en-2-yl)cyclohex-2-enol		1.7		1.5		3.2
19	1H-Pyrrole-2-acetonitrile, 1-methyl-		1.9	2.1			
20	1,4-Methano-1H-Cyclopropa[d]pyridazine, 4,4a,5,5a-tetrahydro-6,6-dimethyl-		1.7				
21	1H-Imidazol-2-amine		2.6	2.9			
22	4-Amino-2-methyl-N-[(5-methylfuran-2-yl)methyl]pyrazole-3-carboxamide		1.2				
23	Piperityl tiglate, trans-		1.7				
24	Normethsuximide, N-trimethylsilyl-		1.2				
25	(R, Z)-2-Methyl-6-(4-methylcyclohexa-1,4-dien-1-yl)hept-2-en-1-ol			1.7			
26	1,3-Cyclohexadiene, 5,6-dimethyl-			1.8			
27	1,3,8-p-Menthatriene				2.2		
28	alpha.-Phellandrene				1.3		
29	Benzene, 2-ethyl-1,3-dimethyl-				2.4		
30	(E)-Atlantone				0.9	0.8	1.1
31	2-(4-Fluorophenoxy)-4-methyl-1,3-thiazole-5-carboxylic acid				1.4		
32	(−)-Indoline-2-carboxylic acid				2.7		
33	3-Methylbut-2-enoic acid, 3,5-dimethylphenyl ester				1.9		
34	3-Decen-5-one				2.0		2.3
35	Acetic acid, cyano-, salicylidenehydrazide					1.8	
36	Cyclopropyl phenylmethanol					2.1	
37	Pinocamphyl tiglate, iso-					2.0	
38	Phenyl tiglate, 2-allyl-					1.9	
39	Piperityl tiglate, cis-					1.7	
40	Ethanone, 1-(1-methylcyclopentyl)-						1.3
41	Benzene, 1-methyl-4-(1-methylpropyl)-						1.1
42	aR-Turmerone						37.0
43	4-(1,5-Dimethylhex-4-enyl)cyclohex-2-enone						1.1
44	Benzene, 1,2,3,5-tetramethyl-						1.8
45	Bicyclo[3.2.1]oct-3-en-2-one, 4-methyl-						3.9
46	Bicyclo[3.1.1]heptan-2-one, 6,6-dimethyl-, (1R)-						2.8
47	2,4-Dimethylpentan-3-yl (E)-2-methylbut-2-enoate						2.3
48	Phthalic acid, decyl 3-phenylpropyl ester						4.3
49	1,1-Propanedicarbonitrile, 1,2-dicyclohexyl-						1.8
50	3-Methyl-2-butenoic acid, 2-methyloct-5-yn-4-yl ester						3.8

Results showed that the extracts obtained through SFE contained higher amounts of tumerone (44.6–54.5%) and curlone (11.5–18.4%) compared to the HD process. The lower yield observed for HD may be attributed to the decomposition of volatile compounds due to the high temperature (~100 °C) applied during extraction (~3 h). Consequently, degradation of volatile compounds may occur leading to lower extraction efficiency compared to the SFE process (8, 35). However, the HD-extracted oil was the only one in which ar-turmerone was identified, and it was present at a high concentration (37%). Literature suggested that ar-turmerone inhibits microglial activation, a property beneficial for the treatment of neurodegenerative diseases (14). Moreover, Chowdhury et al. (38) and Devkota & Rajbhandari (14) reported ar-turmerone contents of 27.8 and 4.3%, respectively, in HD-extracted turmeric oil, which are 1.3 and 8.6 times lower than the values obtained in this study. The composition of turmeric essential oils can vary depending on the origin of the raw material, extraction methods, and process variables (14, 39).

As shown in Figure 2b, the highest tumerone percentage (54.5%) in turmeric oil was obtained under TO conditions (160 bar / 43 °C), whereas T6 had the lowest tumerone content (44.6%). T6 was characterized by a high extraction temperature (50 °C), which reduces oil solubility in supercritical CO₂. In this research results showed that TO and T6 had higher tumerone levels than those obtained through HD in the studies by Chowdhury et al. (34) and Naz et al. (35). Additionally, Figure 2b indicates that the highest curlone percentage (18.4%) in the oil was found in the TO treatment, whereas T6 had the lowest curlone percentage (16%). The curlone obtained in this study were slightly higher than those reported in previous studies (38–40), which documented curlone contents between 10 and 13%.

### 3.2 Ultrasound-assisted extraction (UAE)

Results concerning Box–Behnken (BB) experimental design for the second step (UAE) of the sequential process are presented in Supplementary Table S3.

#### 3.2.1 UHPLC-q-TOF-MS/MS profiling analysis of UAE-extracts

Phytochemical profiling of the polar fractions of *C. longa*, obtained using UAE in the second stage, resulted in the tentative identification of 10 compounds. These identifications were based on high-resolution mass data, MS/MS fragmentation patterns, comparisons with spectral databases (such as HMDB, Metlin, and MassBank), and existing literature. Table 2 presents the phytoconstituents detected via ESI-q-TOF-MS/MS in negative ionization mode, listing retention times (min), molecular formulas, observed [M-H]^−^ ions, mass error (ppm), and characteristic MS/MS fragment ions. Peak 1 was tentatively identified as p-hydroxybenzoic acid, displaying a deprotonated molecular ion [M − H]^−^ at m/z 137.0251. The MS/MS fragmentation produced a diagnostic product ion at m/z 93, corresponding to the loss of CO_2_ [(M − H − CO2)^−^]. The decarboxylation product was also observed in the fragmentation of the precursor ions corresponding to peaks 2 and 3, generating product ions at m/z 123 and m/z 119, respectively. Based on these fragmentation patterns, the compounds were tentatively identified as vanillic acid (peak 2) and p-coumaric acid (peak 3). The product ion observed at m/z 134 in negative ionization mode for peak 4 (ferulic acid) was tentatively assigned to the fragment [M − CO − CH3 − H]^−^, resulting from consecutive losses of CO and CH3 groups. On the other hand, peaks 5–10 were tentatively identified as curcuminoid compounds based on their characteristic MS/MS fragmentation patterns, which showed agreement with previously reported data by Vardhini et al. (41), Jia et al. (42), and Singh et al. (43). Peaks 5 (tR = 8.245) and 9 (tR = 8.970) were putatively assigned as bisdemethoxycurcumin (BDMC) isomers, based on their deprotonated molecular ion [M − H]^−^ at m/z 307 and the characteristics fragments ions at m/z 145, 119, and 117. According to Jia et al. (42), m/z 145 is formed from BDMC keto form through a hydrogen transfer from benzene ring to C-4 and loss of a neutral moiety, in contrast m/z 119 is produced from BDMC enol form. Additionally, further fragmented product ion at m/z 117 is obtained from m/z 145 via the neutral loss of CO. Demethoxycurcumin isomers (DMC) (peaks 6 and 9) showed a [M − H]^−^ at m/z 337 and peak product ions at m/z 175, 173, 145, and 119. As described by Vardhini et al. (41), m/z 175 and 173 serve as diagnostic ions formed by Retro-Diels-Alder cleavage of *β*-diketone or keto-enol bridge in curcuminoids. Peaks 7 and 10 were proposed as curcumin isomers based on fragment ions at m/z 217, 175, and 149. The fragmentation ion at m/z 217 [M − H − 150] − represents the presence of methoxyl groups in vanillyl moieties (43).

**Table 2:** Major phenolic compounds in curcuma extracts by UHPLC-q-TOF-MS analysis.

Peak	t_R_ (min)	Tentative identification	Molecular formula	Monoisotopic mass	*m/z* observed [M-H]^−^	Error (ppm)	MS/MS diagnostic product ions (*m/z*)
1	3.196	*p*-hydroxybenzoic acid	C_7_H_6_O_3_	138.0317	137.0251	4.98	108.0211, 93.0339
2	3.627	Vanillic acid	C_8_H_8_O_4_	168.0423	167.0351	0.70	123.0099, 79.0204
3	4.700	*p*-Coumaric acid	C_9_H_8_O_3_	164.0473	163.0399	−1.04	119.0364
4	5.227	Ferulic acid	C_10_H_10_O_4_	194.0579	193.0505	−0.69	134.0376
5	8.245	Bisdemethoxycurcumin I	C_19_H_16_O_4_	308.1049	307.0981	1.68	145.0150, 119.0365
6	8.371	Demethoxycurcumin I	C_16_H_18_O_8_	338.1154	337.1074	−2.15	175.0254, 145.0150
7	8.440	Curcumin I	C_21_H_20_O_6_	368.1260	367.1200	3.51	175.0252
8	8.970	Bisdemethoxycurcumin II	C_19_H_16_O_4_	308.1049	307.0988	3.96	145.0150, 119.0365
9	9.064	Demethoxycurcumin II	C_16_H_18_O_8_	338.1154	337.1095	4.08	173.0408, 145.0150, 119.0365
10	9.166	Curcumin II	C_21_H_20_O_6_	368.1260	367.1204	4.60	217.0245, 175.0195, 149.0434

**Figure 3:**
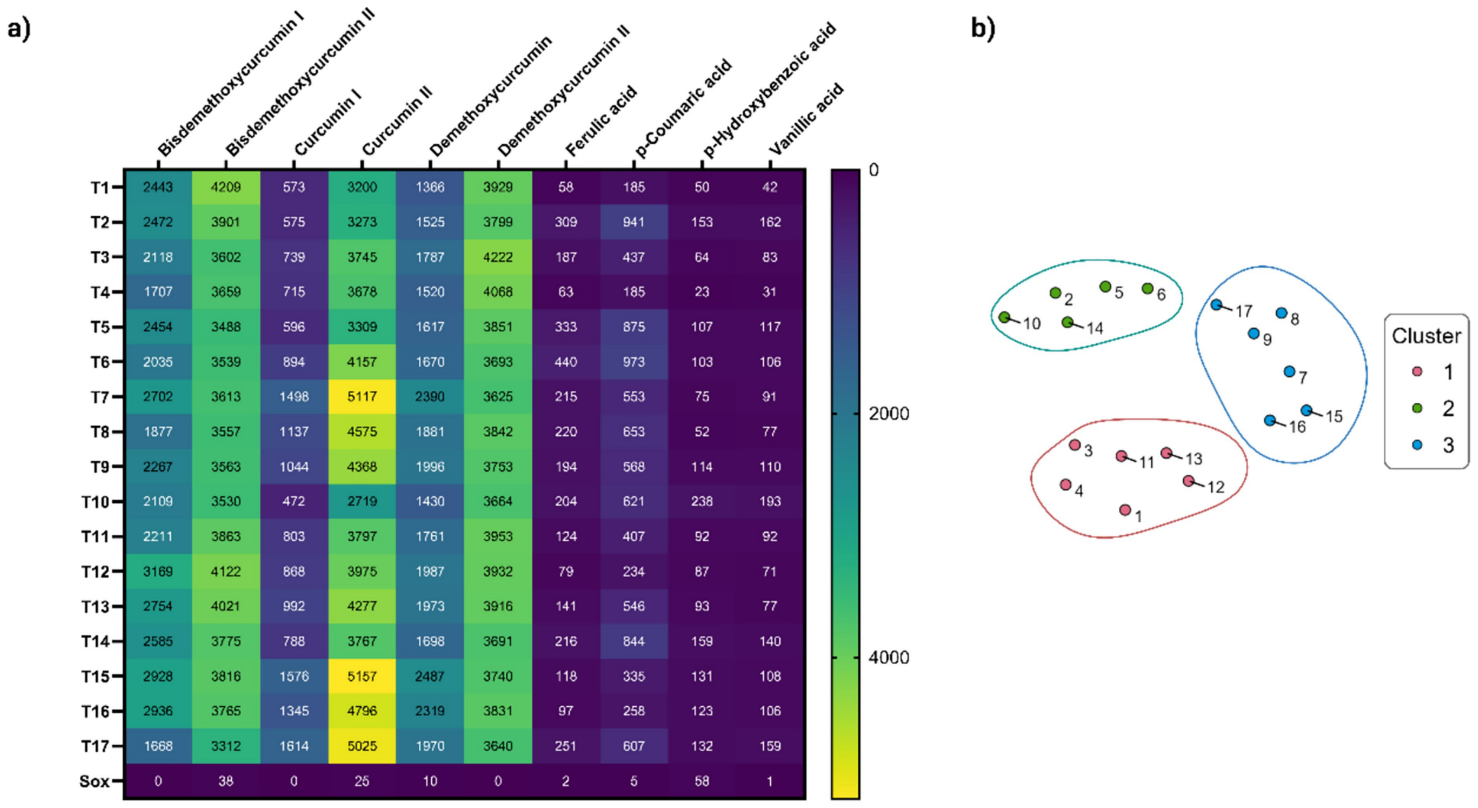
**(a)** Heatmap showing the distribution of the main phenolic components in UAE-extracts (T1-T17) and Soxhlet extract (Sox). Relative abundance of detected compounds (×10^3^ peak area) measured by precursor ion MS are showed in cells. Color gradient indicating concentration levels (yellow = highest, dark blue = lowest); **(b)** t-SNE visualization of Hierarchically Clustered UAE-extracts based on chemical composition profiles. Numbers represent the treatments. Cluster 1 (T1, T3-T4, T11-T13) with high bisdemethoxycurcumin I, curcumin I, and demethoxycurcumin II; Cluster 2 (T2, T5-T6, T10, T14) rich in phenolic acids (e.g., p-coumaric) but low in most curcuminoids; and Cluster 3 (T7-T9, T15-T17) with intermediate phenolic acids and elevated curcumin II/demethoxycurcumin, suggesting a transitional profile.

Phytochemical profiling results were used to compare extracts obtained through UAE and conventional Soxhlet extract (used as reference). For this, a targeted profiling analysis was used based on the characterization of major phenolic compounds (Table 2). The relative abundance (peak area) of each compound (Peaks 1–10) was determined from extracted ion chromatograms (EIC) employing the deprotonated molecular ion [M − H]−. The differentially enriched compounds in the UAE-extracts are shown in the heatmap of Figure 3a. While no qualitative differences were observed between treatments, quantitative variations are evident. Curcuminoids consistently demonstrated higher abundance than phenolic acids across all extracts. Notably, extracts T7 and T15-T17 showed the most pronounced accumulation of curcumin, along with BDMC and DMC isomers. Comparative analysis revealed that the Soxhlet extraction method demonstrated significantly lower recovery efficiency for phenolic compounds relative to UAE technique. In addition, UAE-extracts were grouped according to their phenolic profile, as a result of a hierarchically cluster analysis. Figure 3b displays the t-SNE analysis results, revealing three distinct clusters. Cluster 1 comprises extracts T1, T3-T4, and T11-T13, characterized by: (i) predominant levels of bisdemethoxycurcumin I, curcumin I, and demethoxycurcumin II; and (ii) comparatively lower concentrations of curcumin II, demethoxycurcumin I, and phenolic acids. Cluster 2 (T2, T5-T6, T10, T14) exhibits a distinct phytochemical profile, characterized by: (i) higher phenolic acid content relative to other clusters, with *p*-coumaric acid being particularly abundant, and (ii) reduced levels of all curcuminoids except curcumin I. Cluster 3 groups extracts T7–T9 and T15–T17, occupying a central position in the t-SNE space between the other clusters. This spatial arrangement suggests a transitional role, supported by its intermediate chemical composition: while phenolic acid content is moderate compared to Clusters 1 and 2, the group is distinguished by high levels of curcumin II and demethoxycurcumin.

#### 3.2.2 Characterization of the extracts

Results concerning bioactives extraction (TPC, curcuminoids content), antioxidant activity (DPPH, ABTS) as well as *in vitro* biological activities (AChE Inhibition, Anti-Inflammatory - LOX) are presented in Supplementary Table S3.

#### 3.2.3 Total phenolic content (TPC)

**Figure 4:**
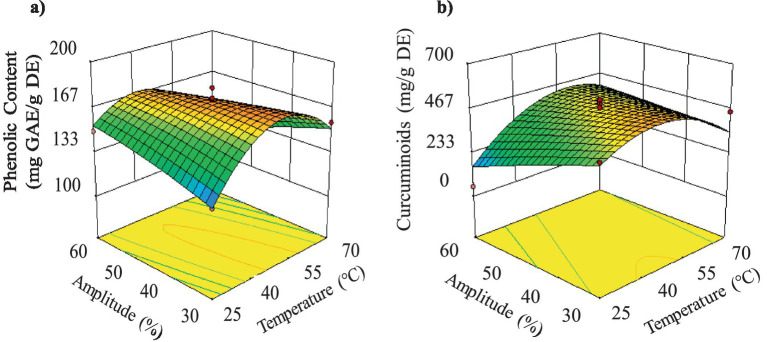
Response surfaces for the UAE stage: **(a)** total phenolic content; **(B)** curcuminoid content.

The results of ANOVA (Supplementary Table S4) showed that the solid/solvent ratio, the interaction between temperature and amplitude, and the quadratic term of the solid/solvent ratio were the variables that had a significant impact (*p* < 0.05) on TPC. *F*-tests’ *p*-values confirmed the statistical significance (*p* < 0.05) for TPC model and no significant lack of fit (*p* > 0.05). Experimental results (Figure 4a and Supplementary Table S3) showed that the highest total phenolic content in the extracts was 179.51 mg GAE/g DE, obtained with treatment T13 with Temperature equal to 47.5 °C (intermediate); Solid/Solvent ratio equal to 1:25 (high) and Amplitude of 30% (lowest). This TPC value was 4.3 times higher than that obtained through Soxhlet extraction (42.03 mg GAE/g DE). Similar results were found in other studies (44). The positive effect of amplitude on TPC can be attributed to higher cavitational intensity forming microbubbles, which collapse intensely, generating physical effects such as cell wall rupture. This leads to a higher extraction yield of the target compounds due to greater solvent penetration (45). Regarding the temperature effect, the literature indicates that moderately high temperatures reduce solvent viscosity while increasing solute diffusivity and solubility, thereby improving extraction. However, a substantial temperature increase during the extraction of turmeric compounds can cause degradation of the target solute (8).

#### 3.2.4 Curcuminoids content

The results of the analysis of variance indicate that only the quadratic term of temperature showed significant effect (*p* < 0.05) in curcuminoid content (Supplementary Table S5). F-tests’ *p*-value show significant lack of fit (*p* < 0.05). A significant lack of fit is an indication that the prediction of the model is not appropriate. A significant Lack of Fit *F*-value of 7.99 can only be caused by noise in 3.65 percent of cases. Finally, the model was considered inadequate for predicting results.

Even if it was not possible to obtain a predictive model, observation of the results presented in Supplementary Table S3 showed that curcuminoid content increased when the temperature ranged between 45 and 60 °C, the solid/solvent ratio was 1:25, and the amplitude was maintained between 30 and 45% (Supplementary Table S3). The maximum curcuminoid contents were determined to be 604.40 and 552.70 mg/g DE, obtained in treatments T1 and T13, respectively. Observations agreed with the response surface (Figure 4b) that allowed describing curcuminoids’ behavior during the UAE process as a function of the Amplitude and Temperature.

Comparing UAE with Soxhlet extraction, the curcuminoid content obtained through UAE was up to 3.3 times higher. This result can be attributed to an enhanced mass transfer caused by acoustic cavitation (44). The implosion of cavitation bubbles on the particle surface leads to cellular structure fragmentation, reducing particle size, increasing surface area, and improving mass transfer (46). The findings of this study align with previous research conducted by Landim Neves et al. (47) and Insuan et al. (44).

#### 3.2.5 Antioxidant activity (DPPH and ABTS)

**Figure 5:**
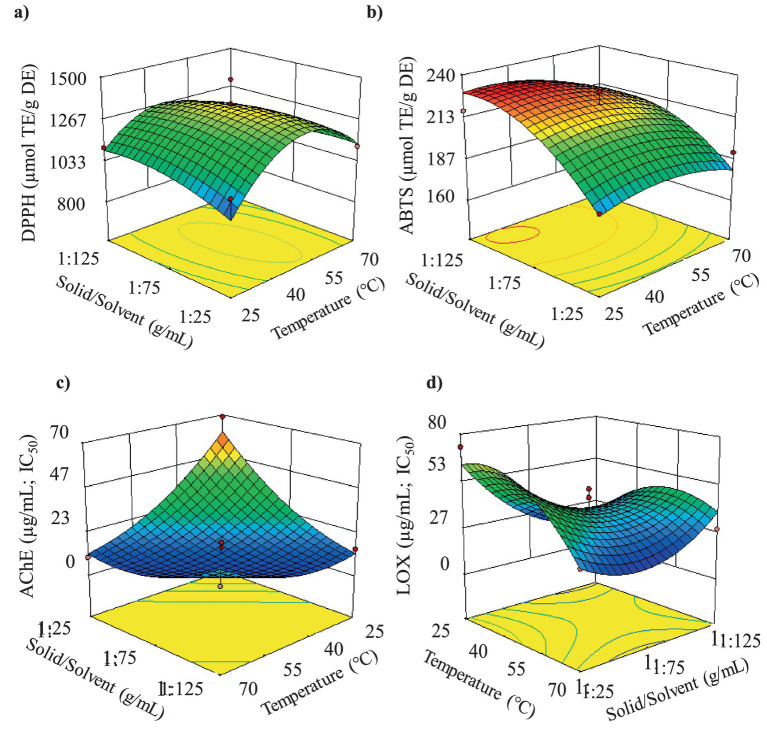
Response surfaces for **(a)** antioxidant activity by DPPH method; **(b)** antioxidant activity by ABTS method; **(c)** neuroprotective activity (AChE inhibition); **(d)** anti-inflammatory activity (LOX inhibition).

The results of the analysis of variance (Supplementary Table S6) indicate that for DPPH only the quadratic term of temperature showed significant differences (*p* < 0.05). Lack of fit showed a non-significant *p*-value of 0.28, which was also the evidence for the adequacy of the model. For ABTS response (Supplementary Table S6), the solid/solvent ratio was the only term that had a significant factor (*p* < 0.05) and non-significant Lack of fit (*p* < 0.05) for the model. The results indicated (Supplementary Table S3) that antioxidant activity determined by ABTS increased as the solid/solvent ratio approached 1:125. Figure 5a shows that antioxidant activity (DPPH) values were higher when temperatures ranged between 40 and 55 °C. Experimental data showed that the highest antioxidant activity by DPPH was obtained at the central point, with 1331.4 μmol TE/g DE (average of 5 central points). Nurcholis et al. (48) reported antioxidant activity (DPPH) values of 92.35 μmol TE/g DE using maceration with prior stirring (30 min at 140 rpm) followed by maceration for 48 h in a dark room. The results from Nurcholis et al. (44) were lower than those obtained in this study. Differences may be attributed to the extraction methods used and the turmeric varieties studied. High antioxidant activity values may be due to the combined effect of total phenolics, total flavonoids, and curcuminoids content (49, 50).

On the other hand, Figure 5b indicates that antioxidant activity determined by ABTS increased as the solid/solvent ratio approached 1:125 and at low temperatures, although as mentioned, the effect of temperature was not significant. The highest antioxidant activity by ABTS (231.71 μmol TE/g DE) was obtained in treatment 7.

Different studies reported that the presence of curcuminoids significantly contributes to antioxidant capacity (11, 51). The antioxidant properties of curcuminoids are attributed to their ability to reduce mitochondrial oxidative stress by enhancing the effects of superoxide dismutase, glutathione, and catalase, which collectively help neutralize free radicals (12, 13). Curcumin acts by reducing lipid peroxidation and increasing glutathione levels in the brain (52, 53).

#### 3.2.6 *In vitro* biological activities

##### 3.2.6.1 Inhibitory activity of acetylcholinesterase enzyme *in vitro* (AChE)

The results of the analysis of variance (Supplementary Table S7) and response surface analysis showed that temperature, solid/solvent ratio, and the interaction between these two variables were the terms that presented significant differences (*p* < 0.05) in the IC_50_ values for AChE inhibition. *F*-tests’ *p*-value show a non-significant lack of fit (*p* < 0.05), this implies that the model can be employed for further analysis.

Results indicated that the extracts with the highest neuroprotective potential originated from treatments using extraction temperatures between 40 and 60 °C and solid/solvent ratios between 1:75 and 1:125. The results of turmeric extracts suggest that they contain bioactive compounds with high neuroprotective potential, as indicated by acetylcholinesterase inhibition assays, which showed IC_50_ values ranging from 5.21 to 68.86 μg/mL (Supplementary Table S3). The treatment that exhibited the highest neuroprotective potential (5.21 μg/mL) was T9, obtained at 47.5 °C of temperature, 1:125 ratio solute/solvent and 60% of Amplitude (Figure 5c). According to the classification by Dos Santos et al. (54) extracts with IC_50_ < 20 μg/mL have high potential, while those with 20 < IC_50_ < 200 μg/mL have moderate potential. Even if it is true that compared to the value obtained for galantamine (0.40 μg/mL), used as a reference inhibitor, our extracts showed between 13 and 172 times lower activity, based on the mentioned classification, all the obtained extracts present high neuroprotective potential, except for the extracts from treatments T12, T8, T1, and T4, which exhibit moderate potential.

These data are comparable to those reported by Ahmed & Gilani (55), who investigated the ability of curcuminoids to inhibit the enzyme AChE. They found that the curcuminoid mixture inhibited AChE in a dose-dependent manner, with an IC_50_ = 19.67 μM. Among the individual components, bisdemethoxycurcumin was the most potent (IC_50_ = 16.84 μM), even surpassing the mixture. It was followed by demethoxycurcumin (IC_50_ = 33.14 μM), while curcumin was the least effective (IC_50_ = 67.69 μM). The authors suggest that the higher efficacy of bisdemethoxycurcumin is due to a structural change (replacement of a methoxy group with a hydroxyl group). Furthermore, tests in the frontal cortex and hippocampus using a fixed dose of curcuminoids (30 μM) confirmed that bisdemethoxycurcumin is capable of crossing the blood–brain barrier and more effectively inhibiting AChE than curcumin, which proved to be the least potent inhibitory compound compared to the original mixture. Despite these findings, the researchers propose that the curcuminoid mixture could serve as a multi-target therapy for addressing Alzheimer’s disease.

Some studies show that curcumin acts as an epigenetic regulator that controls the fate of neural stem cells by decreasing histone protein acetylation. Furthermore, curcumin-mediated neuroprotection involves the reduction of lipid peroxidation, peroxynitrite formation, and an increase in endogenous antioxidant enzyme levels (51, 53, 77). Chen and Decker (56) and Joshi et al. (53) indicate dthat curcumin can be considered an effective neuroprotector due to its ability to efficiently cross the blood–brain barrier as a nanoparticle, thus supporting its therapeutic potential in mitigating various neurodegenerative disorders.

Kalaycıoğlu et al. (57) evaluated the inhibitory potential of the three main compounds, curcumin, demethoxycurcumin (DMC) and bisdemethoxycurcumin (BDMC) that constitute curcuminoids against AChE enzyme, finding that all three have high inhibitory potential. However, bisdemethoxycurcumin exhibited significant AChE inhibitory activity (0.66 μg/mL), which was even 12.6% higher than that of galantamine. Meanwhile, demethoxycurcumin and curcumin showed AChE inhibitory activity of 6.66 and 7.26 μg/mL, respectively. Extracts from *Curcuma longa* rhizomes, obtained using eutectic solvents, exhibited high AChE inhibition percentages (99.24%) and, therefore, high neuroprotective potential Oliveira et al. (11).

Compared to the AChE values for *Ammodaucus leucotrichus* obtained using pressurized ethanol, these showed lower neuroprotective activity values (AChE, IC_50_ = 55.6 μg/mL) and anti-inflammatory activity (LOX, IC_50_ = 39.4 μg/mL) (3). Various studies based on AChE enzyme inhibitory activity demonstrate the potential of different biological extracts. For instance, Ruiz-Domínguez et al. (6) reported that *Durvillaea antarctica* extracts obtained with pressurized liquids had an AChE IC_50_ of 148.62 μg/mL. Additionally, Suárez-Montenegro et al. (4) indicated that *Cyphomandra betacea* extracts, obtained using pressurized liquids (water: ethanol), had AChE IC_50_ values of 97.46 mg/mL of extract.

##### 3.2.6.2 *In-vitro* anti-inflammatory activity (LOX)

The analysis of variance (Supplementary Table S8) and response surface analysis showed that the interaction between temperature - solid/solvent ratio, the interaction between the solid/solvent ratio - amplitude, as well as the quadratic terms of temperature and the solid/solvent ratio, presented significant differences (*p* < 0.05) with a non-significant lack of fit (*p* > 0.05) for LOX inhibition values model.

The results indicated that the extracts with the highest activity against the LOX enzyme were those obtained through treatments performed at temperatures between 25 and 47.5 °C, solid/solvent ratio between 1:75 and 1:125, and amplitudes between 40 and 60% (Figure 5d and Supplementary Table S3). The results of the lipoxygenase (LOX) enzyme inhibitory capacity assays to measure anti-inflammatory activity showed that turmeric extracts had high potential, reporting IC_50_ values between 17.89 and 86.74 μg/mL (Supplementary Table S3).

Previous studies reported that the presence of curcuminoids is associated with anti-inflammatory capacity, which is attributed to a reduction in lipid peroxidation, inhibition of the binding of the transcription factor NF-κB (nuclear factor kappa B) to DNA, leading to a decrease in pro-inflammatory cytokine concentrations, as well as a reduction in circulating inflammatory levels of gamma interferon and inhibition of COX-1 (cyclooxygenase-1 enzyme), COX-2 (cyclooxygenase-2 enzyme), and LOX activities (7, 13, 53, 58). Additionally, curcumin regulates IL-6 (interleukin-6), IL-8 (interleukin-8), tumor necrosis factor-alpha, and COX-2 expressions, which are responsible for the progression of inflammation in the body (7, 52, 53). Subtain et al. (9) evaluated turmeric oleoresin and found that it can exhibit high LOX inhibitory activity, attributed to the presence of ar-turmerone and curlone, which are associated with anti-inflammatory, antioxidant, antimicrobial, and anticancer properties (34).

It is possible to confirm the potential of biological activities using Cell-based methods. These are essential to validate biological activity in a more physiological context, evaluating complex cellular processes and possible toxicity, which brings the results closer to *in vivo* reality. Cell-free *in vitro* methods offer precision, high throughput and control to identify the direct interaction of a compound or extract with a specific molecular target (e.g., enzymes, receptors), being suitable for initial screening (59, 60).

### 3.3 Morphology and FTIR of turmeric

**Figure 6:**
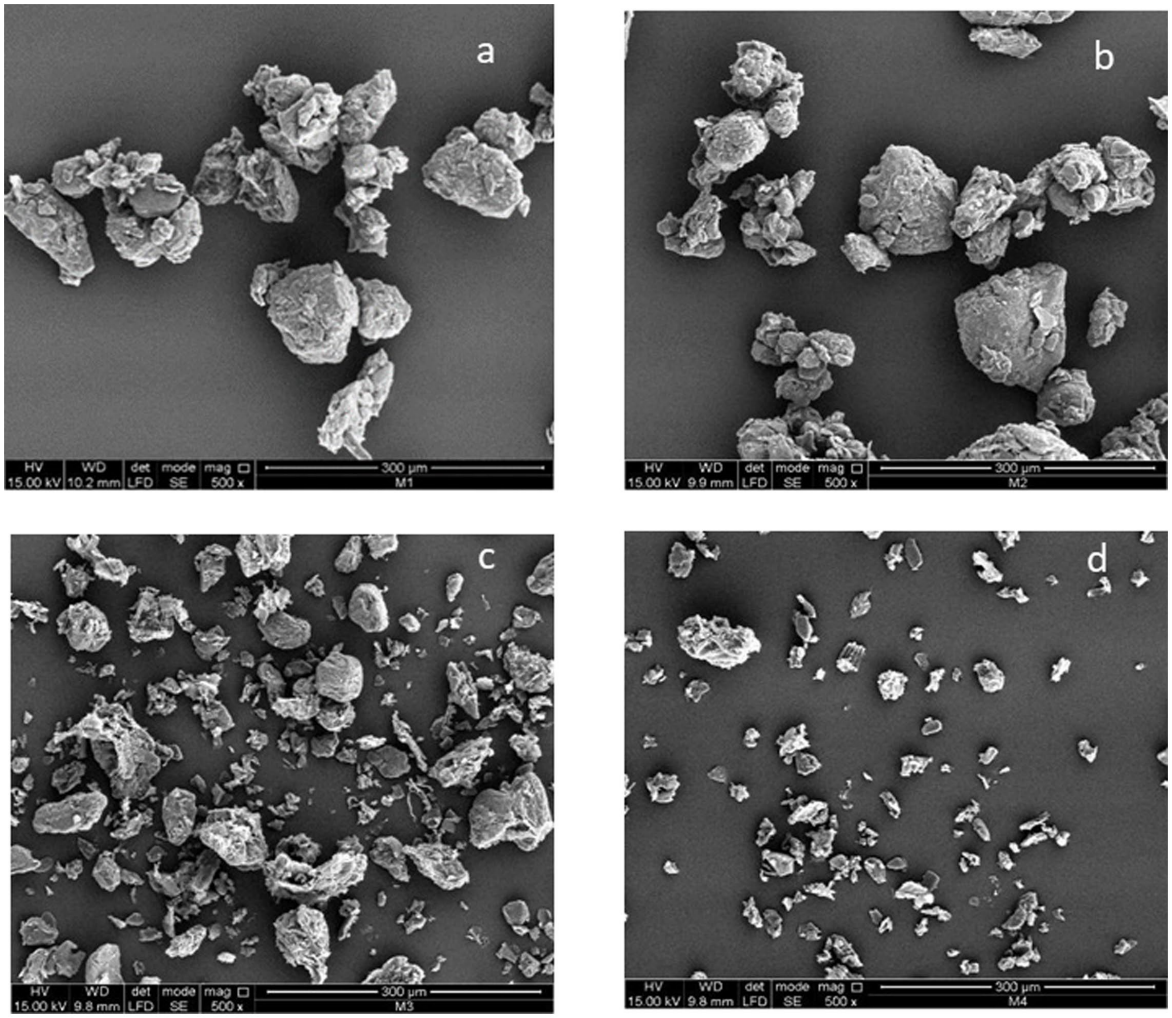
Scanning electron microscopy images of turmeric before and after extraction processes (Magnification 500 x). **(a)** Turmeric powder (Control), **(b)** post-SFE, **(c)** post-UAE, **(d)** post-SFE+UAE.

Figures 6a,b show that no changes occurred in the morphology of turmeric before and after extraction with supercritical CO₂. Similar results were reported by Braga et al. (61), who found that the supercritical extraction process did not cause changes in morphology or in the starch structure of turmeric. Braga et al. (61) considered that this behavior is related to the fact that the pressure did not modify the crystalline structure of turmeric starch and did not alter its degree of gelatinization.

On the other hand, Figures 6c,d show that ultrasound-assisted extraction (UAE) induced changes in the structure of turmeric. This behavior may be attributed to the cavitation phenomenon generated in UAE, in which microbubbles form due to cavitation processes and micro-agitations that collide and generate shock waves, causing damage (ruptures or cracks) in the cell wall (62–64). This results in a reduction in particle size and an increase in surface area, which translates into a higher extraction rate (65).

**Figure 7:**
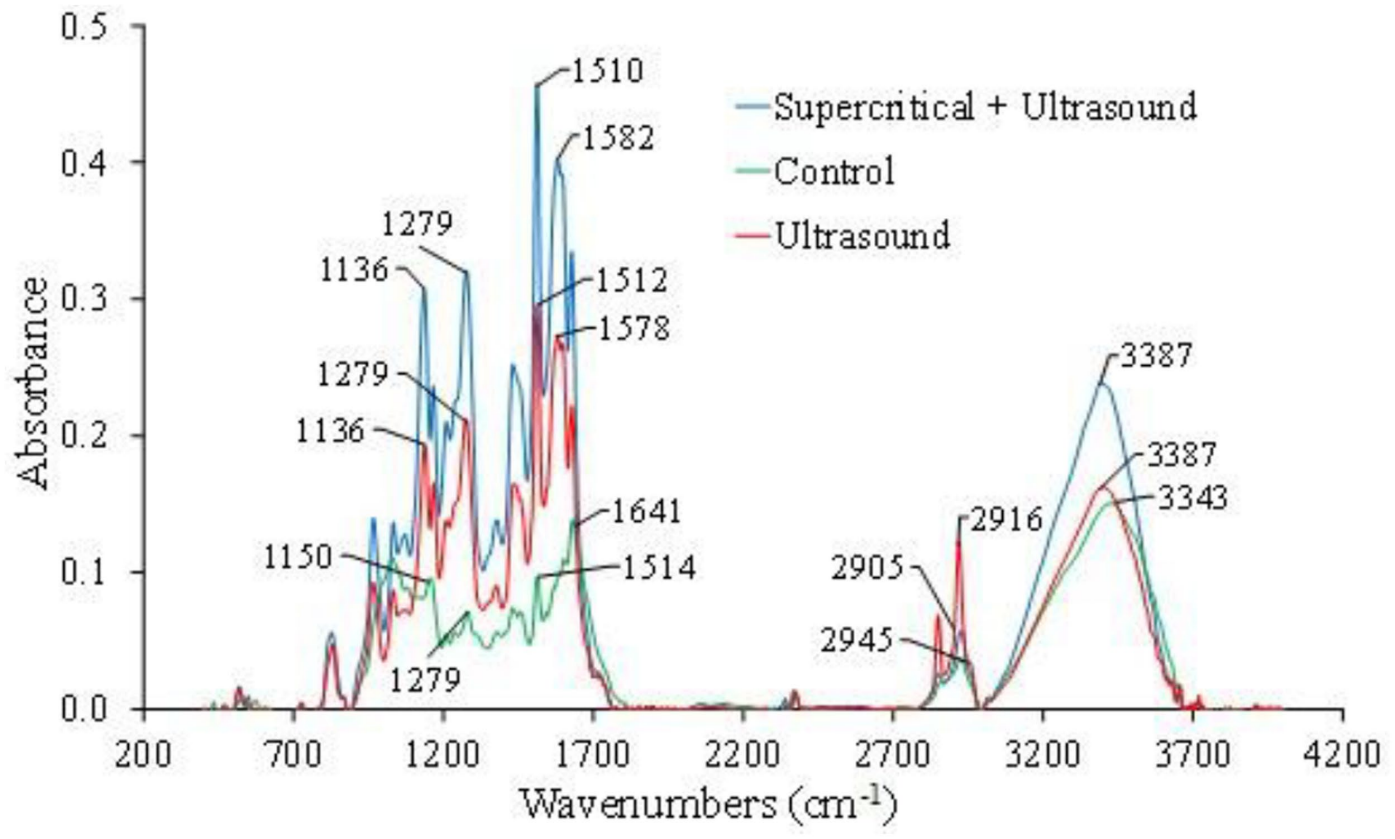
FTIR spectra of turmeric powder: control (unextracted), supercritical fluid + ultrasonic-assisted extraction and ultrasonic-assisted extraction. The control corresponds to the raw turmeric powder material prior to any extraction treatment.

Figure 7 shows that the FTIR spectra of the three samples analyzed in this study exhibited similar trends. This may be due to the fact that the primary structure and composition of the functional groups in turmeric were not altered by the extraction processes studied (47). The obtained FTIR spectra (Figure 7) revealed the presence of turmeric functional groups in different regions. In the band region between 900 and 1,330 cm^−1^, characteristic absorbances of polysaccharides were identified, with a significant presence of carbonyl (C=O) groups in this region. Meanwhile, the band between 1,490 and 1700 cm^−1^ was associated with the OH group of adsorbed water and protein amide (51). On the other hand, in the 2,920 cm^−1^ region, a weak signal (low proportion of compounds) was identified due to vibrations caused by the stretching of the C-H group, including CH, CH₂, and CH₃. Similarly, the functional groups observed in the 3,000 to 3,700 cm^−1^ region were related to the stretching of the O-H group. These last two bands (2,920 cm^−1^ and 3,000 to 3,700 cm^−1^) are characteristic of all polysaccharides, and considering that turmeric contains approximately 69.4 to 81% carbohydrates, the obtained FTIR spectra corroborate this information (13, 52).

The results showed that all three samples presented similar crystalline regions. However, more defined peaks were observed in the samples subjected to extraction treatments. This may be due to the fact that the control sample (turmeric without extraction) contained more amorphous regions than turmeric subjected to extraction using SFE + UAE. The control sample exhibited a broad band with a wider peak pattern because molecules in the amorphous state are more disordered, thus producing more dispersed bands (63).

### 3.4 Stability of nanoemulsions prepared with turmeric extracts

Nanoemulsions were prepared using extracts from treatment T13 obtained at 47.5 °C, 1:25 solid/solvent ratio and 30% of Amplitude (Supplementary Table S3). This treatment exhibited, on average, high concentrations of curcuminoids, a high concentration of total phenolics, and high values for both antioxidant activity (DPPH and ABTS) and *in vitro* biological activities (AChE and LOX inhibition). The stability of nanoemulsion was assessed over 15 days, evaluating them on days 1 and 15 after preparation.

The results of dynamic light scattering (DLS) analysis (Table 3) indicate that the average particle diameters of the extract nanoemulsion remained relatively stable, with values of 136.7 nm on day 1 and 138.3 nm on day 15. The observed increase of 1.17% by day 15 suggests that nanoemulsions maintained stability throughout the analysis period. These results are consistent with those reported by Confessor et al. (18), who found DLS values ranging from 105.70 to 165.40 nm for curcumin nanoemulsion (Supplementary Figure S1). The DLS values obtained in this study indicate that nanoemulsion possess an appropriate size for cellular absorption. This is because particles smaller than 200 nm are ideal for oral administration, as they can more effectively cross the blood–brain barrier (66, 67). These characteristics enhance the bioavailability of turmeric extract, particularly curcuminoids.

**Table 3:** Characterization of turmeric extract nanoemulsions.

Time (day)	DLS (nm)	Zeta potential (mV)	PDI
1	136.7	−34.7	0.212
15	138.3	−31.7	0.215

DLS, dynamic light scattering; PDI, polydispersity index.

Table 3 presents the zeta potential results for nanoemulsion, a measure of the surface charge of the particles and a key indicator of emulsion stability. Zeta potential significantly influences particle stability and the rate at which they aggregate (68, 69). The results of this study (Table 3) showed that the zeta potential (Supplementary Figure S2) decreased by 8.70% on day 15 compared to the value recorded on day 1 (−34.7 mV). Nevertheless, both measurements indicate that the nanoemulsions exhibited good stability, as values above −30 mV are generally considered indicative of good colloidal stability and low coagulation tendency, due to electrostatic repulsion preventing particle aggregation or adhesion (68). Reported zeta potential values for curcumin nanoemulsions vary widely but are consistently negative. Shawir et al. (69) found values ranging from −3.82 to −2.72 mV, whereas Confessor et al. (18) reported values between −33.20 and −32.10 mV.

The results of the polydispersity index (PDI) indicated that the PDI increased by 1.41% on day 15 compared to the value measured on day 1 (PDI = 0.212). The overall PDI of nanoemulsion was 0.21 (Table 3), suggesting a homogeneous distribution of particles (droplets). This characteristic is desirable in pharmaceutical applications as it contributes to system reproducibility and stability. PDI values below 0.3 generally indicate good uniformity in particle size, which is essential for ensuring a uniform release of the active compound and preventing sedimentation (70). Shawir et al. (69) formulated curcumin-based nanoemulsions and obtained PDI values ranging from 0.457 to 0.494, indicating a narrow and desirable particle size distribution (PDI < 0.5), which reduces the effects of gravitational sedimentation. Additionally, Sari et al. (78) prepared curcumin nanoemulsions with lower PDI values (0.273), demonstrating greater stability during storage.

The pH control of nanoemulsions over 15 days showed no substantial changes over time or between different types of nanoemulsions (Table 4). This behavior indicates that turmeric extract nanoemulsions obtained by SFE + UAE remained stable over a 15-day period. In the present study, nanoemulsions maintained a pH between 6.4 and 6.5, consistent with findings reported by Kharat et al. (71), who observed no significant variations over a 15-day period. Similarly, Shawir et al. (69) obtained nanoemulsions with at pH of 6.1 over four weeks. Additionally, Gupta et al. (72) associated pH stability with the high proportion of surfactants and the emulsification process. Marwa et al. (73) indicated that curcumin nanoemulsions should maintain a pH of 6 to prevent curcumin degradation, which occurs when an alkaline pH is reached.

**Table 4:** pH measurements of curcuminoid nanoemulsions versus control.

Time (day)	1	3	9	15
Nanoemulsión extract	6.50	6.49	6.49	6.46
Nanoemulsión control	6.80	6.78	6.76	6.66

Control nanoemulsion formulation: prepared using analytical-standard curcumin (≥75% purity).

## 4 Conclusion

In the work by Degfie Beshah et al. (74), green advanced extraction methods (such as SFE, pressurized liquid extraction, PLE, microwave-assisted extraction, MAE, UAE and enzyme-assisted extraction) and conventional technologies (HD and Soxhlet) were compared in terms of curcuminoids extraction from turmeric. As in the present work, use of conventional technologies provide with lower values of curcuminoids. Results obtained using the mentioned emerging technologies were similar as those obtained in the present work. The combination SFE + UAE provide with higher curcuminoids’ content in the second step and with fractions containing more specific and distinct chemical composition and therefore, more focused biological activities that mainly depend on the chemical class of the compounds present in the fractions. The sequential SFE + UAE process proved to be efficient in obtaining a lipophilic extract by SFE, rich in volatile compounds, and a hydrophilic extract by UAE, rich in curcuminoids and phenolic compounds, using generally recognized as safe (GRAS) solvents, which is consistent with the principles of green chemistry and the Sustainable Development Goals (SDGs). The SFE process demonstrated that the maximum yield of volatile oil (4.0%), with higher contents of tumerone (54.5%) and curlone (18.4%) than those obtained through hydrodistillation, was achieved under conditions of 160 bar and 43 °C. The SFE + UAE sequence enabled the extraction of phenolic-rich (179.51 mg GAE/g DE) and curcuminoids-rich (604.40 mg/g DE) extracts at temperatures not exceeding 47.5 °C. The sequential process enhanced the phenolic content by 4.3 times and the curcuminoids content by 3 times compared to potential, with IC50 values ranging from 5.21 to 68.86 μg/mL. Likewise, these extracts exhibited significant anti-inflammatory activity, as demonstrated by the lipoxygenase (LOX) enzyme inhibition assays. Nanoemulsions of extracts obtained from the sequential process demonstrated improved extracts stability. The results of dynamic light scattering (DLS), zeta potential, polydispersity index (PDI), and pH showed no significant variations, confirming the stability of nanoemulsions.

The Supercritical Fluid Extraction (SFE) combined with UAE presents a viable and environmentally friendly alternative to conventional methods. However, scaling up Ultrasound-Assisted Extraction (UAE) is hindered by the difficulty of achieving uniform cavitation in large volumes, leading to energy inefficiencies and impeding industrial implementation. The next critical step in this research involves conducting *in vivo* studies to confirm neuroprotective activity, for example, using established mouse models.

## Data Availability

The datasets presented in this study can be found in online repositories. The names of the repository/repositories and accession number(s) can be found in the article/Supplementary material.
