# Supplementary material for: Antioxidant, neuroprotective and anti-inflammatory activity of Curcuma longa extracts: from green extraction to nanoemulsion
Source: Front Nutr. 2025 Sep 24;12:1619725. doi: 10.3389/fnut.2025.1619725 (PMC12507333; doi:10.3389/fnut.2025.1619725)
Supplement: Supplementary file 1 [file Data_Sheet_1.PDF]

Supplementary material

Antioxidant, neuroprotective and anti-inflammatory activity of Curcuma longa: From sequential extraction with green technologies to nanoemulsions.

Adriana Marcela Pérez-Munive[1], Hugo Alexander Martínez-Correa[1*], Fabian Parada Alfonso[2], Zully Suárez-Montenegro[3], Diego Ballesteros-Vivas[4], Gerardo Álvarez Rivera[5], Alejandro Cifuentes[6], Elena Ibáñez[6]

[1]Universidad Nacional de Colombia-Sede Palmira-Facultad de Ingeniería y Administración. Carrera 32 No. 12-00, Chapinero, Vía Candelaria, Palmira, Colombia

[2]High Pressure Laboratory, Food Chemistry Research Group, Departamento de Química, Facultad de Ciencias, Universidad Nacional de Colombia, Carrera 30 No. 45-03, 111321, Bogotá D.C., Colombia

[3]Facultad de Ingeniería Agroindustrial, Universidad de Nariño, Ciudad Universitaria Torobajo, San Juan de Pasto, Nariño, Colombia

[4]Departamento de Nutrición y Bioquímica, Facultad de Ciencias, Pontificia Universidad Javeriana, Cra. 7a. No. 43-82, edificio 52 oficina 602, Bogotá, Colombia

[5]Depto. de Química Analítica, Nutrición y Bromatología, Facultad de Química, Universidade de Santiago de Compostela Avda. das Ciencias S/N, Campus Vida, 15782 Santiago de Compostela, Spain

[6]Foodomics Laboratory, Institute of Food Science Research (CIAL) (CSIC-UAM), Nicolás Cabrera 9, 28049 Madrid, Spain

(*e-mail: hamartinezco@unal.edu.co)

Table SM1 Central Composite Design (CCD): Extraction yield of turmeric oil and identified curcuminoid content

Treatment	Variables		Oil Yield (%)	Density CO_2-SC (Kg/m^3)*
	Pressure (bar)	Temperature (°C)		
1	182 (1)	57 (1)	3.27	713.57
2	98 (-1)	43 (-1)	3.07	525.77
3	98 (-1)	57 (1)	0.02	295.24
4	182 (1)	43 (-1)	4.61	804.12
5	140 (0)	50 (0)	4.24	673.03
6	140 (0)	50 (0)	4.74	673.03
7	140 (0)	50 (0)	4.33	673.03
8	140 (0)	50 (0)	4.32	673.03
9	140 (0)	50 (0)	3.45	673.03
10	200 (α)	50 (0)	3.87	784.56
11	80 (-α)	50 (0)	0.03	219.28
12	140 (0)	60 (α)	2.99	561.92
13	140 (0)	40 (-α)	4.00	763.72

* Density data were obtained using the technical equation of state (Span & Wagner, 2003).

Span, R., & Wagner, W. (2003). Equations of State for Technical Applications. I. Simultaneously Optimized Functional Forms for Nonpolar and Polar Fluids. I nternational Journal of Thermophysics, 24(1), 1–39.

Table SM2 Analysis of variance (ANOVA) for supercritical CO_2 oil extraction process

Source	df	Sum of squares	Mean square	F	p-value
Model	5	24.39	4.88	22.93	0.0003*
X_{SC1}: Pressure	1	11.24	11.24	58.81	0.0002*
X_{SC2}: Temperature	1	3.22	3.22	15.15	0.0060*
$X_{SC1}X_{SC2}$	1	1.50	1.50	7.05	0.0327*
X^2_{SC1}	1	8.22	8.22	38.66	0.0004*
X^2_{SC2}	1	0.69	0.69	3.24	0.1149
Error	7	1.49	0.21		
Lack of fit	3	0.91	0.30	2.09	0.2439
Pure Error	4	0.58	0.14		
Total	12	25.88			
S = 0.46					

*Significant (p<0.05). S: standard deviation, df: Degree of freedom F: Fisher t-test.

Table SM3 Box-Behnken Design for Ultrasound-Assisted Extraction of Turmeric Compounds

Exp	Variables			Extraction Yield (%)	Phenolic content Yield (%)	TPC (mg GAE/g DE)	Curcuminoids (mg/g DE)	DPPH (µmol TE/g DE)	ABTS (µmol TE/g DE)	AChE (µg/mL; IC_{50})	LOX (µg/mL; IC_{50})
	T (°C)	Sol/Solv (g/mL)	A (%)								
1	47.5 (0)	1:25 (-1)	60 (1)	11.92	16.97	169.70	604.40	1078.07	167.26	26.88	86.74
2	47.5 (0)	1:75 (0)	45 (0)	11.88	18.11	181.13	399.60	1300.50	214.04	8.63	17.89
3	47.5 (0)	1:125 (1)	30 (-1)	10.81	16.49	164.86	505.50	1305.04	231.71	12.17	85.07
4	25 (-1)	1:25 (-1)	45 (0)	10.69	15.36	153.57	364.70	1086.48	183.02	68.86	72.79
5	47.5 (0)	1:75 (0)	45 (0)	12.08	16.95	169.51	515.50	1355.78	229.60	8.41	23.25
6	25 (-1)	1:125 (1)	45 (0)	12.35	14.49	144.86	469.50	1111.94	217.73	14.42	17.96
7	25 (-1)	1:75 (0)	60 (1)	12.07	14.90	149.02	51.80	946.83	231.71	11.40	23.34
8	25 (-1)	1:75 (0)	30 (-1)	11.36	13.02	130.20	428.10	938.49	210.47	21.39	23.02
9	47.5 (0)	1:125 (1)	60 (1)	12.20	17.28	172.77	494.70	1175.53	227.53	5.21	19.31
10	70 (1)	1:25 (-1)	45 (0)	12.26	15.34	153.35	532.00	1118.40	191.33	9.63	26.64
11	70 (1)	1:75 (0)	60 (1)	23.05	11.79	117.90	389.17	1027.37	205.33	11.33	21.62
12	70 (1)	1:125 (1)	45 (0)	34.28	12.06	120.64	292.10	880.06	207.02	20.69	28.53
13	47.5 (0)	1:25 (-1)	30 (-1)	14.15	17.95	179.51	552.70	1234.58	191.33	9.27	31.55
14	47.5 (0)	1:75 (0)	45 (0)	11.80	17.37	173.74	473.30	1283.79	214.04	18.08	50.30
15	47.5 (0)	1:75 (0)	45 (0)	11.95	17.28	172.75	494.30	1487.87	229.60	15.25	45.39
16	47.5 (0)	1:75 (0)	45 (0)	11.40	15.96	159.64	461.68	1229.06	221.55	11.06	31.35
17	70 (1)	1:75 (0)	30 (-1)	22.42	15.58	155.82	451.90	1167.48	194.28	10.11	29.46
Soxhlet	~78	--	--	22.42	--	42.03	181	--	--	16.84	34.23
Galantamine	--	--	--		--	--	--	--	--	0.40	--

DE = Dry Extract; TE = Trolox Equivalent; GAE = Gallic Acid Equivalent

Table SM4 Analysis of Variance of Total Phenolic Content (TPC) in extracts from the ultrasound process

Source	df	Sum of squares	Mean square	F	p-value
Model	9	56.19	6.24	10.0	0.0031*
X_{US1}: Temperature	1	1.13	1.13	1.8	0.2213
X_{US2}: Solid /Solv	1	3.51	3.51	5.63	0.0495*
X_{US3}: Amplitude	1	0.55	0.55	0.88	0.3786
$X_{US1}X_{US2}$	1	1.45	1.45	2.33	0.1710
$X_{US1}X_{US3}$	1	8.04	8.04	12.88	0.0089*
$X_{US2}X_{US3}$	1	0.78	0.78	1.26	0.2995
X^2_{US1}	1	40.07	40.07	64.20	<0.0001*
X^2_{US2}	1	0.30	0.30	38.66	0.0004*
X^2_{US3}	1	0.21	0.21	0.34	0.5777
Error	7	4.37	0.62		
Lack of fit	3	1.95	0.65	1.07	0.4553
Pure Error	4	2.42	0.61		
Total	16	60.56			
S = 0.79					

*Significant (p<0.05). S: standard deviation, df: Degree of freedom F: Fisher t-test.

Table SM5. Analysis of variance of curcuminoid content in extracts from the ultrasound process

Source	df	Sum of squares	Mean square	F	p-value
Model	9	195500	21727.09	2.83	0.0921
X_{US1}: Temperature	1	15406.27	15406.27	2.01	0.1996
X_{US2}: Solid/Solv	1	10658.00	10658.00	1.39	0.2773
X_{US3}: Amplitude	1	19813.44	19813.44	2.58	0.1523
$X_{US1}X_{US2}$	1	29704.52	29704.52	3.87	0.0900
$X_{US1}X_{US3}$	1	24581.54	24581.54	3.20	0.1168
$X_{US2}X_{US3}$	1	976.56	976.56	0.13	0.7319
X^2_{US1}	1	73021.97	73021.97	9.51	0.0177*
X^2_{US2}	1	25218.22	25218.22	3.28	0.1129
X^2_{US3}	1	202.90	202.90	0.026	0.8755
Error	7	53771.88	7681.70		
Lack of fit	3	46081.18	15360.39	7.99	0.0365*
Pure Error	4	7690.70	1922.67		
Total	16	249300			
S = 87.65					

*Significant (p<0.05). S: standard deviation, df: Degree of freedom F: Fisher t-test.

Table SM6. Analysis of variance of antioxidant activity determined by DPPH and ABTS in extracts obtained by ultrasound

- DPPH

Source	df	Sum of squares	Mean square	F	p-value
Model	9	326900	36317.57	2.75	0.0977
X_{US1}: Temperature	1	1500.70	1500.70	0.11	0.7458
X_{US2}: Solid/Solv	1	252.68	252.68	0.019	0.8938
X_{US3}: Amplitude	1	21818.56	21818.56	1.65	0.2393
$X_{US1}X_{US2}$	1	17397.61	17397.61	1.32	0.2885
$X_{US1}X_{US3}$	1	5509.35	5509.35	0.42	0.5387
$X_{US2}X_{US3}$	1	182.25	182.25	0.014	0.9097
X^2_{US1}	1	223200	223200	16.92	0.0045*
X^2_{US2}	1	11367.21	11367.21	0.86	0.3841
X^2_{US3}	1	27718.28	27718.28	2.10	0.1904
Error	7	92319.96	13188.57		
Lack of fit	3	53547.72	17849.24	1.84	0.2800
Pure Error	4	38772.24	9693.06		
Total	16	419200			
S = 114.84					

*Significant (p<0.05). S: standard deviation, df: Degree of freedom F: Fisher t-test.

- ABTS

Source	df	Sum of squares	Mean square	F	p-value
Model	9	4555.86	506.21	2.91	0.0865
X_{US1}: Temperature	1	252.79	252.79	1.45	0.2672
X_{US2}: Solid/Solv	1	2852.01	2852.01	16.40	0.0049*
X_{US3}: Amplitude	1	2.04	2.04	0.012	0.9168
$X_{US1}X_{US2}$	1	90.44	90.44	0.52	0.4942
$X_{US1}X_{US3}$	1	25.96	25.96	0.15	0.7107
$X_{US2}X_{US3}$	1	98.90	98.90	0.57	0.4754
X^2_{US1}	1	269.51	269.51	1.55	0.2533
X^2_{US2}	1	824.14	824.14	4.74	0.0660
X^2_{US3}	1	46.35	46.35	0.27	0.6216
Error	7	1217.64	173.95		
Lack of fit	3	975.47	325.16	5.37	0.0690
Pure Error	4	242.17	60.54		
Total	16	5773.51			
S = 13.19					

*Significant (p<0.05). S: standard deviation, df: Degree of freedom F: Fisher t-test.

Table SM7. Analysis of variance of the in vitro acetylcholinesterase (AChE) inhibition assay in extracts obtained by ultrasound.

Source	df	Sum of squares	Mean square	F	p-value
Model	9	2983.57	331.51	5.64	0.0164*
X_{US1}: Temperature	1	516.97	516.97	8.79	0.0209*
X_{US2}: Solid/Solv	1	482.83	482.83	8.21	0.0241*
X_{US3}: Amplitude	1	0.44	0.44	0.0075	0.9333
$X_{US1}X_{US2}$	1	1072.56	1072.56	18.24	0.0037*
$X_{US1}X_{US3}$	1	31.42	31.42	0.53	0.4885
$X_{US2}X_{US3}$	1	150.92	150.92	2.57	0.1531
X^2_{US1}	1	279.30	279.30	4.75	0.0657
X^2_{US2}	1	267.42	267.42	4.55	0.0704
X^2_{US3}	1	198.90	198.90	3.38	0.1084
Error	7	411.52	58.79		
Lack of fit	3	339.27	113.09	6.26	0.0543
Pure Error	4	72.25	18.06		
Total	16	3395.09			
S = 7.67					

*Significant (p<0.05). S: standard deviation, df: Degree of freedom F: Fisher t-test.

Table SM8. Analysis of variance of the in vitro lipoxygenase (LOX) inhibition assay in extracts obtained by ultrasound.

Source	df	Sum of squares	Mean square	F	p-value
Model	9	7196.57	799.62	6.50	0.0110*
X_{US1}: Temperature	1	119.04	119.04	0.97	0.3582
X_{US2}: Solid/Solv	1	558.62	558.62	4.54	0.0706
X_{US3}: Amplitude	1	40.91	40.91	0.33	0.5823
$X_{US1}X_{US2}$	1	804.29	804.29	6.53	0.0378*
$X_{US1}X_{US3}$	1	16.65	16.65	0.14	0.7239
$X_{US2}X_{US3}$	1	3657.23	3657.23	29.71	0.0010*
X^2_{US1}	1	1100.62	1100.62	8.94	0.0202*
X^2_{US2}	1	965.10	965.10	7.84	0.0265*
X^2_{US3}	1	38.40	38.40	0.31	0.5939
Error	7	861.63	123.09		
Lack of fit	3	394.59	131.53	1.13	0.4381
Pure Error	4	467.04	116.76		
Total	16	8058.20			
S = 11.09					

*Significant ($p<0.05$). S: standard deviation, df: Degree of freedom F: Fisher t-test.

Day 1

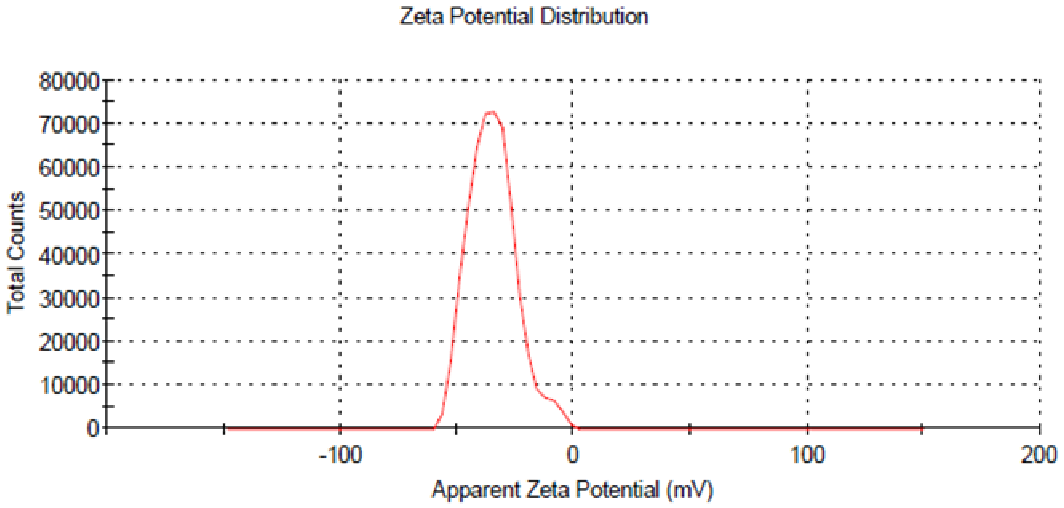

Day 15

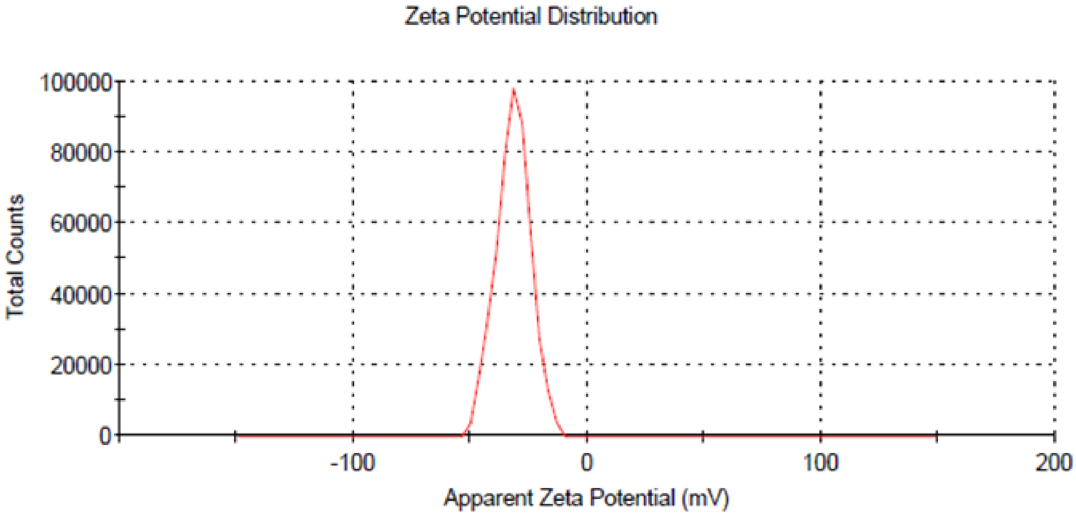

Figure SM1 Zeta potential

Day 1

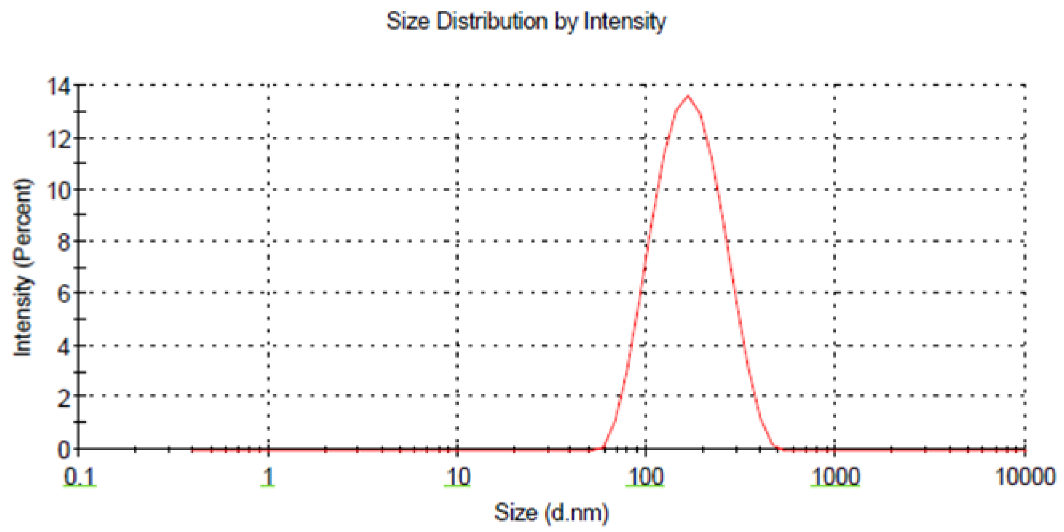

Day 15

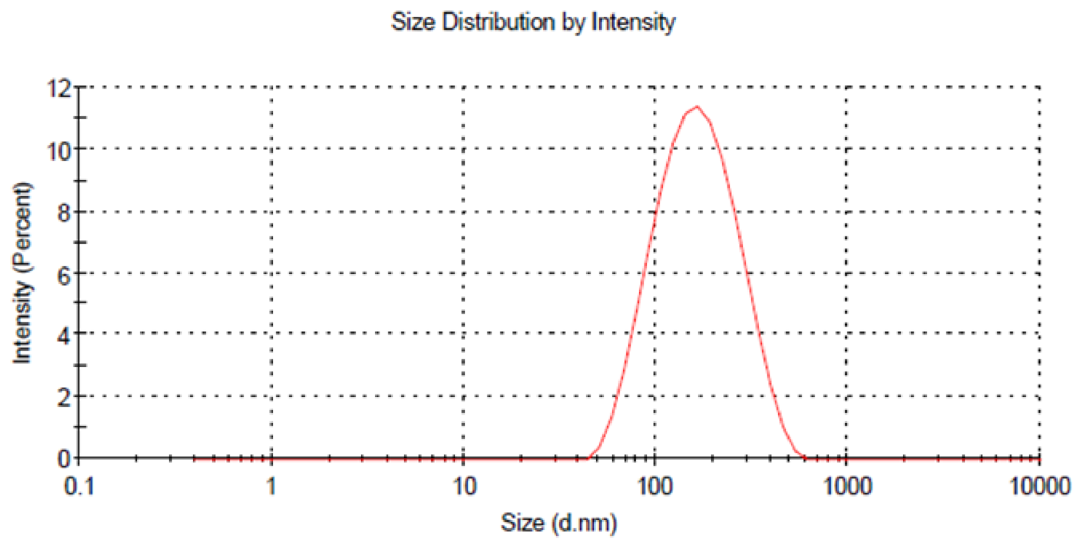

Figure SM2 Size distribution
